# Physiochemical properties of experimental nano-hybrid MTA

**Published:** 2017

**Authors:** V Akhavan Zanjani, K Tabari, SM Sheikh-Al-Eslamian, AN Abrandabadi

**Affiliations:** * Department of Restorative Dentistry, Dental School, Shahid Beheshti University of Medical Sciences, Tehran, Iran; ** Dental Research Center, Research Institute of Dental Sciences, Dental School, Shahid Beheshti University of Medical Sciences, Tehran, Iran

**Keywords:** acidity, calcium, ions, mineral trioxide aggregate, nanoparticles

## Abstract

**Introduction:** Development of new pulp capping agents has paved the way towards the preservation of pulp vitality, which is an important goal in restorative dentistry. This study sought to assess the calcium ion release, pH and setting of mineral trioxide aggregate (MTA) Angelus, an experimental formulation of nano-hybrid MTA containing nano-SiO2, nano-Al2O3 and nano-TiO2 and MTA Angelus plus nano-oxides.

**Methods:** In this experimental study, five specimens from each material were placed in polypropylene tubes and immersed in a flask containing deionized distilled water. The quantity of calcium ions released into the solution from each material was measured at 15 minutes, one hour, and 24 hours by using atomic absorption spectroscopy. The pH of the solutions was measured by using a pH meter at the respective time points. The setting time was also assessed by using a Gilmore needle. Data were analyzed by using repeated measure ANOVA.

**Results:** The quantity of released calcium ions was not significantly different among the groups (P=0.060). All materials were alkaline and the pH at 24 hours was significantly higher than the other two time points in all groups (P<0.001). The experimental group had the shortest and the MTA Angelus had the longest setting time. All materials were alkaline and capable of releasing calcium. Addition of nanoparticles to MTA Angelus significantly decreased the setting time but had no effect on the release of calcium ions or pH.

**Abbreviations:** MTA = mineral trioxide aggregate, VPT = vital pulp therapy

## Introduction

The development of new pulp capping agents has paved the way towards the preservation of pulp vitality, which is an important goal in restorative dentistry. The purpose of vital pulp therapy (VPT) is the maintenance and stimulation of the remaining healthy tissue to promote the healing of the dentin-pulp complex. Suitable candidates for VPT include vital primary/ permanent teeth with full/ partially root formation after traumatic/ carious/ iatrogenic pulp exposure. In addition, adequate blood supply, severity of inflammation, hemostasis, disinfection of exposure/ cavity preparation and coronal seal are important criteria for a successful treatment [**1**,**2**].

Nanotechnology has enabled the production of nanoscale dental materials with improved physicochemical properties. Different nanoscale oxides such as SiO2, TiO2 and Al2O3 nanoparticles have been incorporated into the formulations of dental materials and cements to enhance their physical and mechanical properties and long-term clinical success [**3**,**4**]. These nanoparticles provide greater surface area and higher reactivity and excitability [**4**]. Addition of nano-Al2O3 to cements increases the compressive strength and modulus of elasticity of cements [**5**]. Incorporation of nanoparticles fills the gaps and creates a less porous, dense mass leading to an increase in modulus of elasticity [**5**]. Addition of 3wt% nano-SiO2 to cements was shown to increase the reactivity and compressive strength of 7 and 28 days cements and decreased the size of Portland crystals [**6**]. Nano-TiO2 is also used due to its unique anti-bacterial, self-cleaning and photoelastic properties. 

MTA (Mineral Trioxide Aggregate) is composed of tricalcium silicate, dicalcium silicate, tricalcium aluminate, tetracalcium aluminoferrite, calcium sulfate and bismuth oxide and is available in two forms of white and gray MTA. It has unique properties and provides an optimal seal, which is improved over time. It was first used for the apical seal in retrograde surgeries but at present, it has numerous applications such as the repair of perforations in the pulp chamber floor, repair of furcal perforations, induction of the formation of an apical barrier in immature necrotic teeth and pulp capping in permanent teeth.

Nano-hybrid MTA is a newly invented cement with the base of Portland cement that contains nano-SiO2, nano-Al2O3, nano-TiO2, and microsilica for more favorable physicochemical properties and Bi2O3 for greater radiopacity. The experimental cements was fabricated in the laboratory of Parseh Dental Promotion Center (Yehran, Iran) by Kasra Tabari [**6**]. The manufacturer claims that this cement has optimal properties. However, further studies are required to test the efficacy of this cement. 

In order to induce mineralization, a material must have high pH and should be capable of releasing calcium ions. These capabilities are required for hard tissue healing [**7**]. Calcium ions released from cements react with phosphate ions present in body fluids and lead to the mineralization and formation of hard tissue. MTA was shown to produce deposits resembling hydroxyapatite in both composition and structure in synthetic tissue fluids [**8**]. A high pH is also a requisite for pulp capping agents since high pH is directly correlated with the antibacterial activity of cements, and is critical for the formation of a hard tissue barrier [**9**].

Although the release of calcium ions and the pH of MTA have been the subject of many previous investigations, the comparison with nano-hybrid MTA has not been studied [**10**,**11**]. Moreover, finding a solution to shorten the setting time of these materials, without changing their pattern of calcium ion release, while creating in an alkaline environment, will eliminate a major obstacle against the current use of these materials in the clinical setting.

Considering the need to overcome the shortcomings of the commercially available MTA, this study sought to assess the calcium ion release, pH and setting time of MTA Angelus, MTA Angelus plus nano-oxides and one experimental formulation of nano-hybrid MTA. 

## Materials and methods

The pH, setting time, and calcium ion release of MTA Angelus (Angelus, Londrina, PR, Brazil), MTA Angelus+ nano-oxides and one experimental formulation of nano-hybrid MTA were evaluated in this in-vitro experimental study. The MTT assay was previously used to show the biocompatibility of nano particles and experimental nano-hybrid MTA [**12**,**13**]. A total of 5 specimens were fabricated of each cement with the following compositions: 

- Experimental nano-hybrid MTA: Portland cement+ Bi2O3+ nano silica+ nano alumina+ nano-TiO2+ micro silica+ nano-hydroxyapatite (powder/ liquid ratio: 3/ 1)

- MTA Angelus (powder/ liquid ratio: 3/ 1)

- MTA Angelus+ nano-TiO2 + nano-SiO2+ nano-Al2O3(powder/ liquid ratio: 3/ 1)

Polypropylene tubes with an internal diameter of 1mm and height of 1cm were used for this purpose. Tubes were weighed before and after being filled with cements in order for all the tubes to have the same weight of cement material. All the cements inside tubes weighed approximately 0.05g. Pastes were packed inside polypropylene tubes by using a hand condenser. Excess water at the two ends of the tube was absorbed by paper towel. Cement residues were cleaned from the external walls. To reduce error, five specimens were fabricated of each cement. The specimens were then gently transferred to glass flasks containing 10mL of deionized distilled water and the lids were sealed. To prevent contamination of specimens, glass flasks were washed with 3M nitric acid, thoroughly rinsed with deionized distilled water, and dried in an oven prior to use. Flasks containing specimens were incubated at 37°C with >90% relative humidity. To measure the amount of calcium ions released and pH at 15 minutes and one hour, the five specimens made of each cement were carefully transferred to another flask containing fresh deionized distilled water and after the measurement of the pH and the amount of calcium ions released into the solution, the specimens were returned to the primary flask. The measurements were repeated at 24 hours, but the specimens were put aside and were not returned to the primary flask. The solution inside the flasks was tested to measure the pH and the quantity of calcium ions released. The pH meter (Model 744, Metrohm, Switzerland) was first calibrated for the measurement of pH by using standard solutions with pH values of 4 and 7 and the probe of the device was thoroughly washed with deionized distilled water and dried with paper towel after each use. The pH measurements were made at 28 ± 1°C. 

Atomic absorption spectroscopy (Perkin-Elmer model 1100B, Phoenix, AZ, USA) was used (air/ acetylene flame, calcium cathode lamp) to measure the quantity of calcium ions released. First, a standard calibration curve was drawn by using standard solutions of calcium chloride containing 1, 2, 3, 4 and 5ppm calcium concentrations. Next, 20μL of the 5% lanthanum chloride solution was added to 1mL of each standard solution and the standard samples were injected into the device. The lanthanum chloride solution prevents other ions, especially the phosphate ions, from interfering with the measurements. 100μL of the solution in test tubes was added to 900μL of deionized distilled water to measure the quantity of the calcium ions released; 20μL of lanthanum chloride solution was also added and the samples were injected into the device. The value displayed for each specimen was recorded. The results were calculated with ×10 correction factor [**14**].

The setting time was defined as the duration of time from the mixing of the material until no indent could be made on the specimen surface. A Gilmore needle weighing 456.5g was used for indentation to obtain the final setting time. According to ADA specification 57 and ASTM specification C266-03, for the setting test, the mixtures were transferred to ring molds measuring 10mm in diameter and 2mm in thickness. Three specimens were fabricated in each group of cement. After mixing the materials according to the manufacturer’s instructions, a chronometer (Akai, China) with an accuracy of 0.01 second was adjusted to zero and after the termination of the mixing time, the chronometer was started. After packing the specimens into molds, the surface was smoothed by a metal spatula. The tip of the device (needle measuring 2mm in diameter and 4mm in length) was placed on the horizontal surface of the material for 5 seconds at 30-seconds intervals. The indentation caused by the tip of the Gilmore needle was indicative of unset material. 

Repeated measure ANOVA was applied to compare the pH and release of calcium ions over time in different groups. To compare the setting time of different groups or pairwise combinations of groups, one-way ANOVA and Tukey’s HSD test were used respectively. Type 1 error was considered as α=0.05 and P<0.05 was considered statistically significant. 

## Results

**Table 1** shows the mean quantity of calcium ions released from the cements. Repeated measures ANOVA revealed a significant increase in the release of calcium ions over time (P<0.001). However, no significant difference was noted between the 15 minutes and the 1 hour time points (P=0.98). But, the release of calcium ions at 24 hours was significantly greater than that at the other two time points (P<0.001). The quantity of released calcium ions was not significantly different among the cement groups (P=0.060). Moreover, the interaction effect of group and time was not significant (P=0.315). 

The mean pH of materials is shown in **Table 1**. Repeated measures ANOVA revealed a significant increase in pH over time (P<0.001). Although the pH was not significantly different at 15 minutes and one hour (P=0.392), the pH at 24 hours was significantly different from the other two time points (P<0.001). In this study, pH was not significantly different among groups (P=0.564). The interaction effect of group and time was not significant either (P=0.315).

**Table 1:** The mean and standard error of calcium ion release and pH in different groups

Groups	Ca ions release (ppm) ± SE			pH ± SE		
	15 min	1h	24h	15 min	1h	24h
Nano-hybrid MTA	13.80 ± 0.92	12.80 ± 0.90	24.80 ± 0.86	9.47 ± 0.15	9.94 ± 0.12	10.41 ± 0.10
Angelus + nano oxides	10.60 ± 0.92	10.00 ± 0.90	25.20 ± 0.86	9.68 ± 0.15	9.58 ± 0.12	10.58 ± 0.10
Angelus	10.60 ± 0.92	11.40 ± 0.90	24.80 ± 0.86	9.45 ± 0.15	9.63 ± 0.12	10.48 ± 0.10

The mean and standard error (SE) of final setting time in different cement groups are shown in **Table 2**. One-way ANOVA revealed a significant difference in this regard among the 3 groups (P<0.001). Pairwise comparison of groups using Tukey’s HSD test revealed a significant difference between the experimental group and MTA Angelus (P=0.001), but the difference between MTA Angelus+ nano-oxides and MTA Angelus was not significant (P=0.27) in this regard. The experimental group had the shortest and MTA Angelus had the longest setting time.

**Table 2:** The mean and standard error of the final setting time in different groups

Groups	Final setting time (sec)
Nano-hybrid MTA	14.66 ± 1.45
Angelus + nano oxides	24.33 ± 0.88
Angelus	39.00 ± 2.08

## Discussion

MTA has numerous applications in direct pulp capping, repair of root and furcal perforations and apexification. MTA powder is mixed with sterile water and a moist cotton pellet is placed directly over the applied restoration since the moisture helps setting the material. Following the water sorption, a colloidal gel is formed. The properties of this gel are influenced by factors such as the powder /liquid ratio, method of mixing and amount of air trapped in the mixture, pressure applied for condensation, environmental moisture, type of MTA, pH of the environment, type of liquid mixed with the powder, thickness of material and temperature [**15**-**17**]. Gray MTA has a longer initial and final setting time than white MTA [**18**,**19**]. A longer setting time of white MTA compared to Portland cement is due to the lower sulfur and tricalcium aluminate contents of white MTA [**20**]. Considering the importance of calcium ion release in hard tissue formation and also the necessity of high pH for antibacterial activity, we aimed to assess the calcium ion release and pH of experimental formulations of nano-hybrid MTA in comparison with MTA Angelus and MTA Angelus+ nano-oxides.

Nano-hybrid MTA contains Portland cement, Ca(OH)2, Bi2O3, nano-SiO2, nano-Al2O3, nano-TiO2, nano-hydroxyapatite and micro silica in different weight percentages. The mechanism of action of Ca(OH)2 is directly based on the solubility of calcium and hydroxyl ions and subsequent increase in pH [**21**]. In addition to the creation of an alkaline environment, which has antibacterial properties, hydroxyl ions oxidize free radicals with very high reactivity. They react with different types of biomolecules such as bacterial fatty acids. They inactivate endotoxins and denaturize proteins in root canals. By damaging DNA, they disintegrate the cytoplasmic membrane of bacteria [**22**]. Calcium hydroxide can enhance repair and calcification of the adjacent areas by the distribution of calcium and hydroxyl ions [**23**,**24**]. Nano-hydroxyapatite particles have optimal biological properties and by adding them to the restorative materials, we can benefit from their favorable properties.

Several methods are used for the measurement of calcium ions released such as gravimetry, reverse titration, and polarography. However, atomic absorption spectroscopy was used with a cathodic lamp because in contrast to the gravimetry and reverse titration, this method being able to measure minute amounts of ions. Atomic absorption spectroscopy has high measurement accuracy and is among the most widely used techniques for the measurement of the concentration of elements in solutions [**25**]. In terms of sensitivity, polarography is comparable with atomic absorption spectroscopy; however, the former is time consuming and complex. Similar studies have also used atomic absorption spectroscopy for the measurement of calcium ions released from MTA [**26**]. 

The amount of released ions was measured at 15 minutes, one hour, and 24 hours. The measurement at 15 minutes has not been performed in any previous study. Moreover, ion release was not evaluated cumulatively in our study because long-term storage of specimens in the solution leads to a reduction in the release of new ions because the solution becomes saturated with the ions. The release of calcium ions into solutions is based on the diffusion due to the passive transfer of ions despite the low solubility of the compound. When MTA is mixed with water, it forms calcium oxide, which releases hydroxyl and calcium ions. In the current study, the release of calcium ions increased over time in all groups and the difference in this regard between one hour and 24-hour time points was statistically significant. This finding showed that the release of calcium ions continued throughout the entire course of the study in all groups. The current study results are in line with the findings of Massi et al., from 2011, in terms of the release of calcium ions from MTA Angelus at 24 hours [**27**].

A pH meter was used for the measurement of pH, which is also in accordance with previous studies [**28**]. The release of hydroxyl ions occurs based on the diffusion. Generally, all the groups in our study had an alkaline pH similar to the findings of previous studies [**19**,**21**]. Thus, the possibility of induction of hard tissue formation existed in all groups [**28**]. The hydroxyl ions affect the bacterial cytoplasmic membrane, which is necessary for cell metabolism, growth and division [**29**]. The assessment of the pH changes in all groups revealed an increasing trend over time. The difference in pH in all groups was significant at 24 hours compared to the other two time points (15 minutes and one hour). The pH obtained for MTA Angelus in the study by Duarte et al. was lower than the values obtained in our study [**22**]. Although the method of measurement was similar to that employed in the current study, some factors cannot be easily controlled and the standardization of the method of measurement of MTA properties is difficult. In another study by Torabinejad et al., the pH of MTA Angelus reached 12 within three hours. In their study, samples were fabricated in the form of discs and thus, a greater surface of the material was in contact with water [**28**]. Our findings were in line with the results of Amini Ghazvini et al., and the two studies were also similar in terms of study design and methodology [**14**].

The setting time of cements is important since the optimal properties of materials are obtained after the completion of their setting time. According to EN 196-3 (2005) standard, a minimum amount of water must be used for mixing with the powder. On the other hand, the amount of water must be adequate for a complete hydration. In the current study, as described in other studies [**30**], a Gilmore needle weighting 456.5g was used to calculate the final setting time and mixing was done by one operator for all specimens.

Several factors affect the setting time of MTA, such as the amount of water used for mixing, the process of mixing, and the load applied for packing the cement, the environmental moisture, and temperature. The short setting time of the cement decreases the risk of washout and separation of MTA [**31**]. Many efforts have been made to decrease the setting time such as the addition of calcium chloride [**32**], polymers [**19**] and plasticizers [**33**].

In contrast to ISO 6876:2001 standard for testing the setting of dental cements that recommends the use of gypsum molds, metal molds with standard dimensions were used in the current study due to the interference of the gypsum with the MTA setting, this being in accordance with the studies by Torabinejad et al., and Islam et al. [**26**,**28**]. According to the manufacturer, MTA Angelus is a Brazilian cement that sets within 15 minutes. Based on standard conditions of the study, our results (39.00 ± 2.08 minutes setting time) were not in accordance with the manufacturer’s claim in this regard and had a significant difference. However, our findings in this respect were similar to those of Islam et al., and Massi et al. [**26**,**27**].

The addition of nanoparticles to MTA Angelus significantly decreased the setting time in our study. This difference may be attributed to a greater surface area in the group containing nanoparticles. An increased surface area expedites the reaction of powder and liquid. The results of the current study are in line with those of Saghiri et al.; the only difference was that in their study different elements were used to produce the nano white MTA [**31**]. In our study, the experimental groups containing nanoparticles yielded different results due to the differences in the distribution of elements and their composition. The shortest setting time belonged to the nano-hybrid MTA group, which may be due to the greater hydrophilicity of the Portland cement.

Future studies are required to measure the pH and assess the release of calcium ions over longer periods to find out how long this process lasts. Also, for the measurement of ion release, the phosphate buffer must be used to better simulate the in vivo conditions and assess the behavior of cements in conditions closer to the clinical setting. The addition of fluorapatite to MTA formulation instead of hydroxyapatite should be investigated as well for possible applications in indirect pulp capping due to the fluoride release potential and induction of mineralization.

Within the limitations of this in vitro study, the results showed that all the materials studied were alkaline and were capable of releasing calcium. Changes in the composition of these materials had no effect on the release of calcium ions or the pH. The nano-hybrid MTA group showed properties comparable to those of MTA Angelus. The setting time claimed by the manufacturer of MTA Angelus was lower than the obtained results. Addition of nanoparticles to MTA Angelus significantly decreased the setting time but had no effect on the release of calcium ions or pH. 

**Acknowledgements**

Authors would like to thank the Research Institute of Dental Sciences, Shahid Beheshti University of Medical Sciences, Tehran, Iran.

**Disclosures**

The authors declare that they have no conflict of interest.
